# Innate Immunity in the Central Nervous System: A Missing Piece of the Autoimmune Encephalitis Puzzle?

**DOI:** 10.3389/fimmu.2019.02066

**Published:** 2019-09-10

**Authors:** Robb Wesselingh, Helmut Butzkueven, Katherine Buzzard, David Tarlinton, Terence J. O'Brien, Mastura Monif

**Affiliations:** ^1^Department of Neurosciences, Faculty of Medicine, Nursing and Health Sciences, Central Clinical School, Monash University, Melbourne, VIC, Australia; ^2^Department of Neurology, Alfred Health, Melbourne, VIC, Australia; ^3^Department of Neurology, Melbourne Health, Melbourne, VIC, Australia; ^4^Department of Neurology, Eastern Health, Melbourne, VIC, Australia; ^5^Department of Immunology, Faculty of Medicine, Nursing and Health Sciences, Central Clinical School, Monash University, Melbourne, VIC, Australia

**Keywords:** autoimmune encephalitis, innate immunity, microglia, monocytes, epilepsy, neuroimmunology, blood brain barrier

## Abstract

The autoimmune encephalitides are a group of autoimmune conditions targeting the central nervous system and causing severe clinical symptoms including drug-resistant seizures, cognitive dysfunction and psychiatric disturbance. Although these disorders appear to be antibody mediated, the role of innate immune responses needs further clarification. Infiltrating monocytes and microglial proliferation at the site of pathology could contribute to the pathogenesis of the disease with resultant blood brain barrier dysfunction, and subsequent activation of adaptive immune response. Both innate and adaptive immune cells can produce pro-inflammatory molecules which can perpetuate ongoing neuroinflammation and drive ongoing seizure activity. Ultimately neurodegenerative changes can ensue with resultant long-term neurological sequelae that can impact on ongoing patient morbidity and quality of life, providing a potential target for future translational research.

## Introduction

Central nervous system (CNS) autoimmunity is a rapidly advancing field, with significant recent advances in our knowledge of the underlying mechanisms of disease. However, there remains significant gaps in our knowledge, particularly in the genesis of autoimmunity within the CNS and the interaction between the innate and adaptive arms of the immune response. While Multiple Sclerosis (MS) remains the prototypical, and most common, autoimmune CNS disorder, autoimmune encephalitis is a useful disease to further investigate the intersecting processes of the immune response for a number of reasons. First, it has a dramatic onset with clear markers of immune etiology. Second, it affects a broad spectrum of neuronal networks. Third, it has demonstrated the potential for serious long-term sequelae in the form of drug-resistant seizures and cognitive or psychiatric morbidity. The adaptive immune system contribution has been the main focus of investigation into this group of disorders, as exemplified by auto-antibody identification. The innate immune system contribution has been less well-investigated, but it is potentially also important and will be the focus of this review.

## Innate Immune Dysfunction in CNS Autoimmune Diseases

### Blood Brain Barrier Dysfunction

The blood brain barrier (BBB) forms part of the initial defenses of the CNS. BBB permeability can be altered by several factors including inflammatory molecules such as interleukin-1β (IL-1β), tumor necrosis factor-α (TNF-α), C-C motif chemokine receptor-2 ligand (CCL-2), and interleukin-17A (IL-17A) (1).

The main mechanism by which TNF-α mediates BBB disruption is via internalization of tight junction proteins on endothelial cells. This is mediated by upregulation of the downstream pro-inflammatory gene transcription regulator nuclear factor kappa-B (NFkB) (2). These proteins, such as claudin-5, occludin, and zona occludens 1 (ZO-1) prevent transcellular diffusion of molecules and cells (1).

IL-1β contributes to BBB permeability in three major ways. First, it induces expression of matrix metallopeptidase-9 (MMP-9) and vascular endothelial growth factor (VEGF) in endothelial cells, glial cells and monocytes/macrophages that act to degrade tight junction proteins (3–5). Second, IL-1β induces expression of hypoxia-inducible factor-1α (HIF-1α) and VEGF-A, contributing to BBB permeability and increased angiogenesis (4). Third, secreted IL-1β also alters the location of CXCL12 expression in CNS endothelial cells from the basolateral BBB membrane to the luminal surface, contributing to BBB permeability to leukocytes (6).

Experimental autoimmune encephalomyeltis (EAE) is an animal model of CNS autoimmunity and neuroinflammation. Early on in the course of EAE monocyte-derived macrophages produce IL-1β. This can then induce CNS endothelial cells to secrete molecules such as granulocyte-macrophage colony-stimulating-factor (GM-CSF) and granulocyte-colony-stimulating-factor (G-CSF) (7, 8). These factors are important for the recruitment and activation of immune cells (7, 8). In the EAE model, GM-CSF, and G-CSF encourage the differentiation of infiltrating monocytes into antigen presenting cells that can then interact with CD4^+^ cells (9). Mice with the GM-CSF receptor gene deleted only in CCR2^+^ monocytes are more resistant to initiation of EAE. Conversely constitutive GM-CSF secretion by polyclonal T cells results in infiltration of the CNS with myeloid cells (10).

One pathway that is important to innate cell activation and production of inflammatory cytokines is mediated by a family of receptors called Toll-like Receptors (TLRs). Lipopolysaccharides (LPS) and various environmental toxins can act as pathogen-associated molecular patterns (PAMPs), or native molecules such as ATP as damage-associated molecular patterns (DAMPs), to stimulate TLRs found on C-C motif chemokine receptor-2 (CCR2) expressing monocytes (11, 12). Resultant activation of various intracellular signaling-cascades leads to the production and release of pro-inflammatory cytokines.

**Figure 1:**
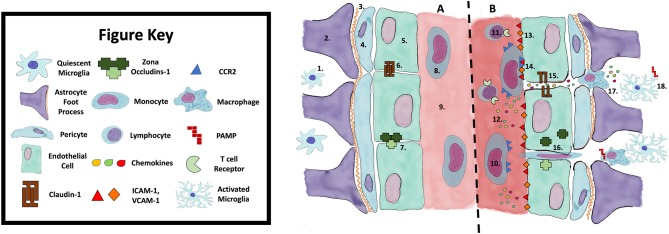
Blood Brain Barrier Function **(A)** and Dysfunction **(B)**. **1**. Quiescent Microglia; **2**. Astrocyte foot process; **3**. Basement membrane; **4**. Pericyte; **5**. Endothelial cell; **6**. Claudin-1; **7**. Zona occludins-1; **8**. Monocyte; **9**. Vessel lumen; **10**. Activated monocyte; **11**. T-lymphocyte; **12**. Chemokines; **13**. ICAM-1, VCAM-1; **14**. Monocyte initiating diapedesis; **15**. Breakdown of tight junctions; **16**. Infiltrating monocyte; **17**. Macrophage; **18**. Activated microglia via DAMP/PAMP. DAMP, Damage associated molecular pattern; PAMP, Pathogen associated molecular pattern; ICAM-1, Intracellular adhesion molecule 1; VCAM-1, Vascular cell adhesion molecule.

Recruitment and activation of these CCR2^+^ monocytes appears to be an important step in neuroinflammation. For example, CCR2 deficient mice exposed to hypoxic-reperfusion injury demonstrate less BBB permeability and smaller infarct size/brain oedema compared with wild type mice (13). The molecule responsible for recruiting CCR2^+^ monocytes, CCL2, also potentially has additional effects on endothelial cells. CCL2 can cause internalization of occludin and claudin-5 (14) within these cells, affecting tight junction integrity. The recruitment of CCR2^+^ monocytes via IL-1β and GM-CSF may play a role in amplification of the pro-inflammatory response, subsequent BBB dysfunction and enhanced interaction between the innate and adaptive immune systems. The contributors to BBB dysfunction are highlighted in Figure 1.

### Innate Cells and Autoimmunity

Innate cells involved in the inflammatory cascade in the CNS include infiltrating monocytes, macrophages, neutrophils as well as the resident microglia.

#### Microglia

Microglia are specialized glial cells found in the CNS that have a “macrophage like” function. They are responsible for the maintenance of the CNS environment as well as a local immune response to injury or infection. Resting microglia exist in a ramified state and are constantly monitoring their environment via processes (15). Activated microglia then alter their morphology and gene expression, allowing them to perform both pro-inflammatory and anti-inflammatory functions (16). Activation of microglia can occur in a number of ways. Microglial activation is strongly linked to extracellular ATP (17). Microglia also express mRNA for TLRs 1–9. *In vivo*, however, TLR3 and 4 are upregulated in inflamed brain tissue (18). Activation of TLRs induce pro-inflammatory cytokine production and expression of MHC-I and MHC-II molecules (19). Other pro-inflammatory cytokines such as CCL2 activate microglia and drive inflammation (20).

Activated microglial cells are an important component of the neuroinflammatory process in the development of MS and subsequent disease progression. Nodules of microglia are found in abundance in normal appearing white matter (NAWM) in tissue autopsy specimens in MS patients (21). These microglia express nicotinamide adenosine dinucleotide phosphate (NADPH) oxidase, a marker of the production of toxic reactive oxygen species (ROS) and a feature of activated microglia (21). There is also a spatial association between inflammation and the presence of microglia in these specimens (21). Some other surface markers of activated microglia, Major Histocompatibility Complex Class II (MHC-II) and CD11c, are seen early in EAE prior to overt infiltration of peripheral immune cells (22).

Activated microglia perform a number of functions in the inflamed CNS. Microglia express CCL2 (the ligand for CCR2), and this expression is upregulated in animal models of demyelination (23) indicating a key role in promoting innate immune cell infiltration. Microglia are also linked to neurodegeneration in CNS autoimmunity. High levels of microglial activation, as measured with [C11]PK11195 positron emission tomography (PET) ligand, in NAWM in MS are associated with brain atrophy and an increasing disability as measured on the Expanded Disability Status Score (EDSS) (24).

Activated microglia can have dual functions, either promoting or decreasing inflammation. TGF-β has been demonstrated to induce microglia to produce anti-inflammatory molecules and down-regulate pro-inflammatory molecules (23). TGF-β injected into a pure neuronal cell culture has protective effects against excitotoxicity (25). In mouse organotypic culture, TNF-α secreted by microglia has similarly been shown to protect neurons from excitotoxicity and promote remyelination (26). In a microglial-hippocampal organotypic coculture, microglia expressing M-CSF are able to decrease NMDA mediated neurotoxicity (27). Similarly, in animal models of neurological disease the neuroprotective role of microglia has been reported. In a mouse model of cerebral ischaemia microglial depletion resulted in a larger infarct size, increased levels of inflammatory compounds, increased immune cell infiltration and increased cell necrosis (28). This was primarily mediated by astrocyte overactivity in the absence of the protective effects exerted by microglial cells (28).

In EAE the inhibition of microglial activation with a tetracycline antibiotic (minocycline) results in an attenuated disease course (29). There is also emerging evidence for minocycline in the prevention of recurrent CNS inflammation (relapse) after a first demyelinating event (30). In this trial patients with clinically isolated syndrome (CIS) who were treated with minocycline had a lower risk of conversion to clinically definite MS. It should be noted that minocycline has other anti-inflammatory properties aside from supressing microglial activation that may play a role in ameliorating EAE or inflammation noted in CIS (31). Inhibition of microglial activation can also be achieved through more targeted methods. Modified rat models utilizing a thymidine kinase suicide gene linked to CD11b reduce the number of activated microglial cells in EAE mice (32). These mice also demonstrate an attenuated clinical course (32).

A number of MS disease modifying medications (DMT) appear to have activity against microglia that may play a role in their efficacy. Aside from its action on lymphocyte trafficking Fingolimod also decreases pro-inflammatory cytokine production and increases the production of neuroprotective molecules produced by activated microglia (33). The immunomodulating small molecule Glatiramer acetate induces an anti-inflammatory profile in microglia and promotes phagocytic activity (34). Another MS disease modifying therapy, Interferon- β, also appears to mediate its protective effect through myeloid cells (35). Mice with selective type-1 interferon receptor in myeloid cells develop severe disease with increased mortality. Conversely selective type-1 interferon receptor knockout in lymphocytes had no effect on the disease course (35).

#### Infiltrating Myeloid Cells

Notably in EAE it appears that the macrophages driving the inflammatory process in demyelinating lesions are actually derived from infiltrating monocytes rather than resident microglia (36, 37). Resident microglia show lower expression of pro-inflammatory genes as compared with these infiltrating macrophages (38). An elegant study in EAE used distinct cell surface markers for resident microglia (CX3CR) and infiltrating monocytes (CCR2) in combination with morphological analysis with electron microscopy and gene expression profiles to examine the role of these myeloid cells (39). It demonstrated that the infiltrating myeloid cells adopted a pro-inflammatory role within the demyelinating lesions. Conversely the resident microglia were far more inert and adopted a more homeostatic role (39).

Infiltrating CCR2^+^ (classical) monocytes appear to be a major monocyte subtype involved in altering BBB permeability and are seen in a number of other models of CNS injury and neuroinflammation. CCR2^+^ monocytes accumulate in brain lesions in traumatic brain injury (TBI) (40). CCR2 knock out mice with a focal TBI demonstrate smaller lesion cavity sizes (40). CCR2 antagonism in focal TBI in mice decreases CCR2^+^ monocyte/macrophage accumulation, which was found to be associated with improvements in cognitive outcomes (14, 41). Monocytes have been shown to migrate to the CNS in hypoxic-ischemic injuries as well as in animal models of amyloid plaque related neurodegeneration (42). In EAE the absence of CCR2^+^ monocytes decrease disease severity (43), indicating a role for CCR2^+^ monocytes in both CNS neuroinflammation and autoimmunity. However, as with microglia, the role of these infiltrating monocytes can be pleiotropic. In a mouse model of spinal cord injury (SCI) inhibition of CCR2^+^ monocyte infiltration into the CNS resulted in chronic microglial activation and delayed clinical recovery (44). The infiltrating myeloid cells in this model had a suppressive effect on pro-inflammatory microglia, providing an anti-inflammatory environment (44).

The other predominant infiltrating myeloid cell in CNS autoimmunity is the neutrophil (45). Neutrophils are early phase effector cells that produce a variety of pro-inflammatory factors such as IL-1β, IL-6, TNF-α as well as ROS (45). In EAE neutrophils appear to play a role in the development of disease, particularly in BBB dysfunction (46). Neutrophils have been shown to infiltrate the CNS in the pre-clinical phase of EAE (47). Depletion of neutrophils prior to disease onset ameliorates disease progression (48). This is not seen with depletion after disease onset or at the clinical peak of disease (48). This suggests an important role of neutrophils in the initial phase of the disease. Similar studies in EAE have demonstrated that BBB dysfunction is spatially related to neutrophil infiltration into the brain (46). Interestingly depletion of neutrophils also diminishes monocyte or microglia maturation into antigen presenting cells (APCs) expressing HLA-DR (49), which may indicate another important role in driving early CNS autoimmunity and neuroinflammation.

### Initiation of the Adaptive Immune Response

One potentially important, yet poorly understood, role of these innate cells in CNS autoimmunity is in stimulating the proliferation and maturation of autoreactive lymphocytes. In the EAE model APCs expressing endogenous antigens promote differentiation of antigen-specific lymphocytes into specific lineages (50). The interaction with T cells appears to play a central role in the initiation of the adaptive response in CNS autoimmunity. MS has long been considered a T cell mediated disease, supported by the presence of activated T cells in active, demyelinating plaques in a large neuropathological study of MS biopsies and autopsy specimens (51). The T cells in MS are thought to be activated by CNS APCs presenting CNS autoantigens, although no specific antigen has been identified (52). The presence of clonally expanded populations of MHC II restricted T cells that are preferentially activated in EAE induction (51) supports the concept of target antigens that activate specific TCRs and induce cellular proliferation.

B cells are also increasingly considered to play a major role in CNS autoimmunity (53). The involvement of B cells in the pathogenesis of MS is supported by the presence of CSF specific oligoclonal immunoglobulins in up to 90% of MS patients (54). Brain tissue specimens from MS patients also demonstrate immunoglobulin and complement deposition in areas of CNS demyelination, indicating B cell antibody production (55). However, as discussed previously, there is no clear antigenic target. Further supporting the role of B cells in MS is the high efficacy of anti-CD20 monoclonal antibodies in preventing relapses in MS and controlling disease progression (56). However, there have also been failures in B cell targeted treatment. Atacicept, a molecule targeting B cell activation factors, actually increased the risk of relapses when used in MS patients (57). It is unclear why these B cell directed treatments have such disparate clinical effects, but does suggest that a potential sub-population of B cells may play a protective role in MS.

B cells may also play a role as APCs in MS. Peripheral B cells from patients with MS have upregulated MHC II expression (58). Higher levels of co-stimulatory molecules are also seen on B cells within the CNS (59, 60). Ablation of MHC II molecules on B cells in mice causes resistance to EAE development (61). Activated B cells also drive T_h17_ responses due to secretion of IL-6 (62, 63) and the absence of B cell secreted IL-6 reduces disease severity in EAE (64). A recent study examining auto-proliferating auto-reactive lymphocyte populations in the peripheral circulation of patients with MS demonstrated the importance of the interaction between the B cells and T cells to maintain activation and proliferation (65). Pertinent to the interaction between the innate and the adaptive immune responses, B cells also play a key role in promoting the ongoing pro-inflammatory response by myeloid cells due to secretion of GM-CSF (66).

## An Emerging CNS Autoimmune Disease: Autoimmune Encephalitis

### Autoimmune Encephalitis Overview

The autoimmune encephalitides (AIE) are a collection of heterogeneous disorders characterized by immune mediated inflammation of the brain parenchyma and disruption of neuronal circuitry (67). Due to the variation in anatomical and functional locations within the CNS that can be affected, these disorders can present with a broad range of symptoms ranging from fevers and headaches to neuropsychiatric disturbance, movement disorders (dystonia/dyskinesia), seizures, cognitive impairment, autonomic dysfunction and sleep-wake cycle disturbance (67). Both individually and as a group, AIE are a relatively rare condition with a measured incidence of 0.8–2/100,000 per year in Europe, and a similar incidence of 1.2/100,000 in the United States of America (68). This is comparable with the incidence of infectious encephalitis (1.0/100,000) (68). Notably this incidence is more than 2-fold greater than in the preceding 10 years, likely reflecting increased recognition and improved diagnostic tests.

### Autoimmune Encephalitis: A Clinical Syndrome

Diagnosis of AIE relies on the recognition of a clinical syndrome together with serological testing. This is supported with identification of inflammation on ancillary investigations. This can include cerebrospinal fluid (CSF) testing looking for pleiocytosis and/or elevated protein, or neuroimaging. MRI brain imaging utilizing gadolinium contrast can demonstrate oedema or increased blood brain barrier permeability. Resultant neuronal circuitry dysfunction can also be identified on electroencephalogram. Due to a reliance on serological testing (and potentially representing some differences in pathophysiology) AIE can be further divided into three sub-groups: (1) a subtype defined by antibodies directed at intracellular targets, (2) a subtype defined by antibodies directed at cell surface antigens, and (3) a further subtype without identifiable antibodies (“seronegative” disease).

The subtype defined by antibodies directed against intracellular antigens are largely paraneoplastic disorders. Despite many similarities to the other subtypes of AIE, this group typically demonstrate poor response to immunotherapy if the underlying neoplastic process is not treated (69). These syndromes and their neoplastic associations are summarized in Table 1. However, a detailed discussion of these specific disorders is outside the scope of this review, but has been covered in a recent publication by Rosenfeld and Dalmau (70).

**Table 1:** Intracellular antibody associated AIE subtypes.

**Antibody**	**Clinical phenotypes**	**Common associated malignancies**
Hu (ANNA1)	Encephalomyelitis, sensory neuronopathy, cerebellar syndrome, limbic encephalitis	Small cell lung carcinoma (SCLC)
Ri (ANNA2)	Ataxia, opsoclonus myoclonus, brainstem encephalitis	Breast, SCLC
Yo (PCA1)	Cerebellar syndrome/degeneration	Ovarian
CV2 (CRMP5)	Sensorimotor neuropathy, retinopathy, optic neuritis, cerebellar syndrome, limbic encephalitis	SCLC, Thymoma
Amphiphysin	Stiff person syndrome, encephalomyelitis, limbic encephalitis	Breast, SCLC
Ma2	Limbic encephalitis, brainstem encephalitis, refractory seizures, myelopathy	Testicular Seminoma
SOX1	Lambert-eaton myaesthenic syndrome, limbic encephalitis	SCLC
Titin	Myaesthenic syndrome	Thymoma
Recoverin	Acute/subacute painless vision loss (Retinopathy)	SCLC, Thymoma
Zic4	Cerebellar syndrome/degeneration	SCLC
Tr (DNER)	Cerebellar syndrome/degeneration	Hodgkins lymphoma

The cell surface antibody associated AIE are a more recently identified entity. Anti-N-methyl-D-aspartate Receptor (NMDAR) antibody associated AIE, characterized in 2007 (71, 72) has a typical clinical syndrome characterized by neuropsychiatric disturbance including psychosis, catatonia, hypersomnia or insomnia, movement disorder (dyskinesia and dystonia) and dysautonomia (73). It is most commonly found in younger females and associated with an ovarian teratoma in 24% of cases (74). Identification of NMDAR antibody associated AIE has led to a search for other novel CNS auto-antibodies. Thus far this research has been fruitful in the identification of a number of clinical syndromes associated with antibodies directed to other cell surface antigens (Table 2).

**Table 2:** Cell-surface Antibody associated AIE subtypes.

**Antibody**	**Clinical phenotypes**
N-methyl-D-aspartate Receptor (NMDAR)	Dyskinetic movements (esp. orofacial), psychiatric symptoms, dysautonomia, catatonia associated with ovarian teratoma
Leucine-rich, Glioma Inactivated 1 (LGI-1)	Limbic Encephalitis, rapid onset dementia, memory impairment, FBDS
Contactin Associated Protein 2 (CASPR)	Limbic encephalitis, neuromyotonia
Gamma-Aminobutyric acid B (GABA_B_)	Refractory seizures, limbic encephalitis
α-amino-3-hydroxy-5-methyl-4-isoxazolepropionic acid (AMPAR)	Refractory seizures, limbic encephalitis, amnestic syndrome
Dipeptidyl-peptidase-like Protein (DPPX)	Gastrointestinal hyperexcitability, limbic encephalitis
glycine receptor	Progressive encephalomyelitis and rigidity with myoclonus (PERM) syndrome, hyperekplexia
Metabotropic Glutamate receptor 1 (mGlu1)	Cerebellar syndrome
Metabotropic Glutamate receptor 5 (mGlu5)	Limbic encephalitis associated with Hodgkin's lymphoma
IgLON5	Sleep disorder, parkinsonism, cognitive dysfunction
Adenylate Kinase 5	Limbic Encephalitis
Gamma-Aminobutyric acid A (GABA_A_)	Refractory seizures

Unsurprisingly, the seronegative subtype is the most difficult to diagnose due to the lack of an “identifiable” antibody in the blood or CSF. A number of these patients have likely non-pathogenic antibodies to antigens such as glutamate decarboxylase (GAD) and Thyroid peroxidase (TPO) (75) which may represent an underlying tendency to autoimmunity. Ancillary investigations supportive of CNS inflammation remain an important criterion for diagnosis (76). Despite the absence of a currently identifiable specific antibodies, this patient cohort appears to have good functional improvement after immunotherapy (77). However, studies on “seronegative” AIE may be potentially confounded by the possible inclusion of undiagnosed viral encephalitides.

### Treatment Targets

#### Investigation and Management of Neoplasms

In AIE syndromes that are associated with neoplasms a prompt and thorough search for a neoplasm is warranted (78). This is particularly vital in subtypes with antibodies directed at intracellular targets but is also true of the other subtypes. It is thought that the tumor provides an antigenic focus that drives the immune response (79, 80). The tumor either expresses an intracellular antigen abnormally on the cell surface, or the antigen becomes exposed during cell necrosis (79, 80). This appears to drive a cell mediated response against neural cells expressing that same antigen (79, 80). The antibodies themselves are not thought to be pathogenic, but rather a biomarker of this immune response. Once the tumor has been identified, removal or treatment of the neoplasm is important to decrease the activity of the immune response and may even ameliorate the syndrome altogether (69). Immunotherapy alone in these patients is unlikely to be successful in the long-term (69).

#### Immunotherapy

The mainstay of treatment in AIE is immunotherapy. First line agents for treatment of AIE includes either monotherapy or combination high dose corticosteroids, intravenous immunoglobulin (IVIg) or plasmapheresis (81). Second line therapy may include cyclophosphamide and/or Rituximab (82). In the rare event of treatment failure, IL-6R antagonists (Tocilizumab) (83) and in some instances proteasome inhibitors (Bortezomib) (84) have been used. The need for longer term “maintenance” immunosuppression is uncertain. In patients with relapsing or refractory disease, longer term “maintenance” treatment with mycophenolate mofetil, azathioprine, ciclosporin or methotrexate has been used (85). This broad range of at least partially effective immunotherapies hints at a potentially complex underlying immunological pathophysiology.

## Autoimmune Encephalitis: Current Evidence of Immunological Dysfunction

### Neuropathology

Neuropathology can often provide an insight into potential effector mechanisms. In AIE pathology provides further evidence for its immune mediated nature, but also highlights its complexity. Multiple small immunopathological studies in AIE (86–93) have demonstrated a variety of pathological changes; these are summarized in Table 3. While minimal consistency is seen amongst these studies, broadly there is a predominance of perivascular lymphocyte infiltration with antibody or complement deposition in cell-surface antibody mediated AIE and proliferation of innate immune cells (microglia and macrophages) across all subtypes.

**Table 3:** Histopathological studies in AIE.

**Histopathological seriesReferences*Tissue*(Number of patients)**	**NMDAR AIE**	**VGKC AIE**	**Seronegative AIE**
Bien et al. (86)*Brain biopsies*(NMDAR = 3, VGKC = 4)	Complement deposition, minimal lymphocyte infiltrate		—
Tuzun et al. (87)*Autopsy*(NMDAR = 2)	Perivascular lymphocyte infiltrate (plasma cells), IgG1 antibody deposition, microglial (CD68) proliferation, atrophy	—	—
Martinez-Hernandez et al. (88)*Brain biopsies and Autopsy*(NMDAR = 5)	Perivascular plasmablasts, no antibody deposition	—	—
Okamoto et al. (89)*Autopsy*(Seronegative = 3)	—	—	Microglial (CD68) proliferation, atrophy
Park et al. (90)*Autopsy*(VGKC = 1)	—	Microglial (CD68) proliferation	
Filatenkov et al. (91)*Autopsy*(NMDAR = 1, post-treatment)	Microglial activation and proliferation. CD3 lymphocyte parenchymal infiltration. Occasional CD20 lymphocyte.	—	—
Khan et al. (92)*Autopsy*(VGKC = 1)	—	Microglial activation. Perivascular CD20 lymphocyte infiltration.	—
Camdessanche et al. (93)*Brain Biopsy*(NMDA = 1)	Perivascular CD20 lymphocyte infiltration	—	—

*NMDAR, N-methyl-D-aspartate Receptor; AIE, Autoimmune Encephalitis; VGKC, Voltage-gated Potassium Channel*.

### Immune Profiles in Autoimmune Encephalitis

There are few comprehensive studies examining the immune cell profile in acute AIE subtypes. CSF flow cytometry performed on two patients with NMDAR antibody associated AIE demonstrated increased CD19^+^ cells with no change in T cell populations compared to patients with non-inflammatory neurological disorders (NIND) (94). A much larger study involving 60 patients with NMDAR antibody associated AIE identified an expanded population of IL-17 producing CD4^+^ T cells (T_h17_ cells) on CSF flow cytometry (95). Another small study examining CSF flow cytometry in 3 partially treated patients with GABA_B_ antibody associated AIE demonstrated increased populations of CD19^+^, CD138^+^, CD4^+^, and CD8^+^ lymphocytes (96). Interestingly in this small series those with activated CD8^+^ cells (measured by presence of HLA-DR) had poorer neuropsychological outcomes than those with activated CD4^+^ cells (96). These studies did not examine innate cells.

A retrospective study of AIE patients with cell-surface antigen targeted antibodies demonstrated an increase in the neutrophil-to-lymphocyte ratio (NLR) on a standard full blood examination as compared with healthy controls (97). Additionally the NLR was positively associated with poor functional outcomes (as measured on the modified Rankin Scale or mRS) in the AIE patients (97). Neutrophils are early responders in the innate immune system, and persistent neutrophil proliferation may be an indication of dysregulation of the pro-inflammatory cascade that extends to the CNS.

### Cytokines in Autoimmune Encephalitis

There are a number of cytokine or chemokine biomarkers in AIE that could provide a clue to immunopathogenesis. Multiple studies in NMDAR antibody associated AIE have shown an increase in CSF CXCL-13 (98), IL-6, IL-17, CXCL-10, IL-1β (95, 99–101), serum IL-2 (102), and, in some but not all studies, CSF B Cell Activating Factor (BAFF) and a proliferation-inducing ligand (APRIL) compared with patients with NIND (103, 104). In comparison the CSF of patients with viral encephalitis typically have increased levels of IL-1β, IL-6, TNF-α, interferon-γ, APRIL, and BAFF (101).

A number of these cytokines are associated with B cells and plasma cells. CXCL13 is a B cell chemoattractant that was demonstrated in CSF of patients with NMDAR antibody associated AIE. Furthermore, decreasing levels correlated with treatment benefit. CXCL13 is known to be produced by monocytes and microglia (105). BAFF and APRIL are B cell activation molecules. In one cohort of AIE patients CSF BAFF and APRIL levels correlated with functional outcomes. Conversely another study comparing NMDAR antibody associated AIE with viral encephalitis noted no elevation of BAFF and APRIL in the CSF (103, 104). AIE patients with antibodies to cell surface proteins have higher CSF levels of interferon-γ, IL-17, IL-12, and IL-23 compared with AIE associated with intracellular antigens (106). These are T cell and more specifically T_h1_ and T_h17_ associated cytokines.

There is also evidence for innate immune system activation in AIE. A recent study identified higher levels of IL-6, pentraxin-3 (part of an innate pro-inflammatory cascade, produced after TLR activation), CD40L and IL-17A in the CSF of patients with NMDAR antibody associated AIE (107). A study examining patients with autoimmune epilepsy presenting with new-onset refractory status epilepticus found elevated levels of IL-6, TNF-α, IL-2, and IL-4 in the CSF, and elevated levels of IL-6 and TNF-α in the periphery (108). Interestingly, treatment with a monoclonal antibody targeting the IL-6 receptor resulted in improvement in seizure activity in 86% of the patients and normalization of the cytokine levels (108).

Other potential AIE biomarkers not directly part of the immune cascade that are consistent with the inflammatory and neurotoxic nature of the immune dysregulation in AIE include Cystatin C and uric acid. Cystatin C levels in the CSF of patients with NMDAR antibody associated AIE are lower during acute disease and improve with treatment (109). Cystatin is suggested to be an anti-inflammatory cytokine and may play a role in neuronal protection through the autophagy pathway (109). Serum uric acid levels similarly seem to decrease in patients with acute NMDAR antibody associated AIE and increase after treatment (110). Uric acid can act as an anti-oxidant and this may reflect increased oxidative stress (110) with inflammation and innate immune cell activation.

### Autoimmune Encephalitis as an Antibody Mediated Disease

The role of antibodies in AIE remains controversial, however there is growing evidence of their pathogenicity in a number of AIE subtypes. The best studied subtype with regards to pathogenicity is NMDAR antibody associated AIE. Serum from patients with NMDAR antibody associated AIE applied to rat hippocampi causes internalization of the NMDA receptors and selectively decreased NMDA neuronal currents as measured by whole cell voltage clamp recordings (111). Rats infused intraventricularly with CSF from individuals with NMDAR antibody associated AIE developed reversible behavioral and memory problems (112). Although the potential presence of other bioactive molecules within the CSF could have confounded these findings, the same changes in NMDAR expression and NMDA mediated currents can be seen in the presence of recombinant NMDAR antibodies alone (113). In humans, NMDAR antibody CSF titres correlate with disease severity and successful treatment (114), also suggesting a potential role in the pathogenesis of this disease.

## Potential Role of Innate Immune System Dysfunction in Autoimmune Encephalitis

### Pathogenesis: BBB Dysfunction, Cellular Recruitment and Antigen Presentation

As we have highlighted in other CNS autoimmune disorders, innate immune cells can perform a number of important functions. (1) They act as the “first line of defense” against pathogens, (2) they perform antigen processing and presentation, (3) they release bioactive factors that can result in BBB dysfunction, and (4) they have the capacity to recruit other immune cells to the CNS. Due to this myriad of functions the innate immune response is highly likely to play an important role in the pathogenesis of AIE.

The possibility of an initial event causing BBB changes is especially convincing in the well-established association between Herpes Simplex Virus-1 (HSV-1) encephalitis and NMDAR antibody associated AIE (115). Patients who are initially diagnosed as HSV-1 encephalitis based on polymerase chain reaction testing may develop another encephalitic illness approximately 4–8 weeks after recovery, marked by the presence of NMDAR antibodies and typical phenotypic features of the latter disease (115). A recent report suggests a similar sequence in a case of GABA_B_ antibody associated AIE (116). In epidemiological studies, there is also a higher incidence of non-encephalitic HSV-1 infections in patients with NMDAR antibody associated AIE as compared with controls (117).

HSV-1 encephalitis produces a pro-inflammatory CNS environment. Mouse microglial cells have been shown to produce high amounts of TNF-α, IL-1β, IL-6, CCL2 during HSV-1 infection. This occurs via TLR2 expressed on microglial cells (118). This microglial activation and cytokine production could drive upregulation and infiltration of innate cells such as monocytes into the CNS, lead to BBB dysfunction as well as recruitment of adaptive immune components such as B cells to produce antibodies. This provides the ideal CNS environment for the genesis of CNS autoimmunity.

The temporal pattern of cytokine production in AIE patients is also informative regarding changes in BBB permeability and potential recruitment of innate cells, followed by recruitment of adaptive immune cells. In a documented case of post HSV-1 NMDAR antibody associated AIE there were three consecutive phases of immune-related molecules seen. First there was an initial spike of pro-inflammatory cytokines in the CSF including IL-1β, TNF-α, interferon-γ and CCL2, as well as CXCL10 and CXCL13, during the HSV-1 phase (119). This first phase suggests both innate and adaptive immune infiltration with BBB dysfunction, consistent with a viral encephalitis. This peak had subsided 2 weeks post first diagnosis. The second phase was characterized by a second peak of CXCL10, CXCL13, and CCL2 in the CSF during the prodromal period (19 days post diagnosis of HSV-1 encephalitis). During this phase there was no NMDAR antibody detected in the CSF. This second phase suggests largely innate immune activation. At the onset of neurological symptoms (day 31), the CSF NMDAR antibody levels peaked while cytokine/chemokine levels dropped off aside from CXCL10 and CCL2 (119). During this third phase the humoral response appears to be playing a large role. These three phases of immune profiles generate a potential hypothesis for the pathogenesis of AIE. First an initial insult altering BBB permeability allowing microglial activation and monocyte/macrophage infiltration. This would then instigate further neuroinflammation, recruitment of B and T cells and subsequent antibody production.

**Figure 2:**
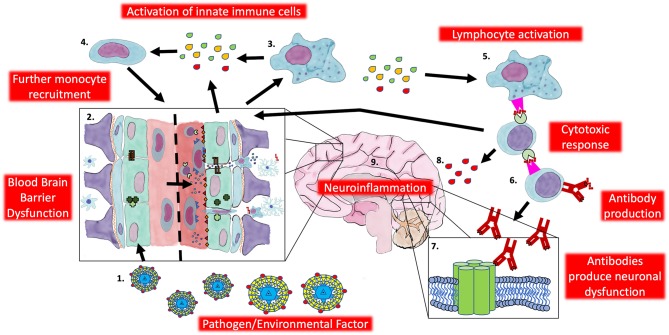
Potential innate contribution to the pathogenesis of autoimmune encephalitis. **1**. An exogenous factor (i.e., herpesvirus) infiltrates the CNS resulting in **2**. blood brain barrier dysfunction and infiltration of innate and adaptive cells. **3**. Activated innate cells (i.e., Macrophages and microglia) release pro-inflammatory cytokines (IL-1β, TNF-α, Interferon-γ) and chemokines (CCL2) to **4**. recruit more innate cells and contribute further to BBB dysfunction. Pro-inflammatory cytokines and chemokines also recruit lymphocytes and innate cells act as **5**. antigen presenting cells to activate T cells and initiate a specific response against neuronal antigens. **6**. T cells interact and activate B cells to produce an antibody response **7**. directed against neuronal targets resulting in neuronal dysfunction while **8**. directing a cytotoxic response against neuronal tissue (and contributing to the pro-inflammatory cascade) resulting in **9**. neuroinflammation. CNS, Central nervous system; IL-1β, Interleukin-1β; TNF-α, Tumor necrosis factor-α; CCL2, C-C motif chemokine ligand 2; BBB: Blood brain barrier.

There is other indirect evidence for this hypothesis. In patients NMDAR antibodies appear in the CSF before the serum (120). In the animal models of NMDAR antibody mediated neuronal injury discussed previously, the changes in NMDA receptors on rat neurons only occurred when the patient serum was infused into the ventricles or when using ApoE knockout mice [who have impaired BBB function (121)] compared with wild type mice (122). This highlights the requirement for an initial neuroinflammatory event to drive the adaptive response. A potential mechanism for pathogenesis is proposed in Figure 2.

Imaging studies have also demonstrated the importance of neuroinflammation and altered BBB permeability in AIE. Imaging of patients with NMDAR antibody mediated AIE utilizing arterial spin labeling MRI techniques early during the disease process have demonstrated focal areas of hyperperfusion prior to T1 or T2 MRI changes (123). This suggests early increased BBB permeability early in the disease course, prior to parenchymal neuroinflammation. Fluorodeoxyglucose (FDG)-PET neuroimaging in multiple studies in various subtypes of AIE demonstrates areas of both hypermetabolism and hypometabolism (124). Anatomical patterns are associated with specific subtypes such as NMDAR and LGI-1 antibody associated AIE (125–128). While the areas of hypometabolism may be related to receptor signaling loss due to antibody binding, the areas of hypermetabolism (as with the hyperperfusion in the case of MRI) could indicate excitotoxicity due to seizures, neuroinflammation or early increased BBB permeability.

Finally, there is genetic evidence indicating a significant role for antigen presentation, the intersection between the innate and adaptive immune response, in AIE. A number of genetic studies looking at HLA associations in different subtypes of AIE have identified some common haplotypes of both MHC-I and MHC-II molecules (129–132); these are summarized in Table 4. While the preponderance for certain MHC-II haplotypes suggest an important role for the interaction between professional APCs (B cells, macrophages, dendritic cells) and CD4^+^ T cells, the MHC-I molecule associations implicate a role for CD8^+^ mediated immune responses.

**Table 4:** HLA haplotypes associated with AIE subtypes.

	**NMDAR AIE (129, 132)**	**LGI-1 AIE (129–131)**
MHC-I	HLAB*07:02	HLA-B*57:01 HLA-B*44:03 HLA*C*06:02 HLA-C*07:06
MHC-II	HLA-DRB1*16:02	HLA-DRB*07:01 HLA-DQA1*02:01 HLA-DQB1*02:02

*NMDAR, N-methyl-D-aspartate Receptor; AIE, Autoimmune Encephalitis; LGI-1, Leucine glioma-inactivated-1*.

### Persistent Neuroinflammation and Antibody Independent Sequelae

A second potential role for the innate immune system is propagation of the neuroinflammatory state and therefore ongoing symptoms such as seizures.

There has been increasing awareness that there is both an increased tendency for seizures in autoimmune neuroinflammation and that seizures themselves can produce a pro-inflammatory state. Several studies have demonstrated that the pro-inflammatory cytokines IL-1β, IL-6, TNF-α modulate susceptibility to limbic seizures in rodent models of temporal lobe epilepsy (133). These cytokines are also upregulated within the CNS during seizures along with markers of monocyte activation (CD86, HLA-DR, CD14^+^CD16^−^) and T cell activation (CD25, CD69, CTLA-4, and HLA-DR) (134). One study examining status epilepticus (SE) induced in rats with kainic acid (KA, a commonly used molecule for inducing seizures in animal models) demonstrated infiltration of blood derived monocytes expressing CCR2 (135). These cells interact with resident microglia and increase levels of IL-1β (135). Prevention of monocyte infiltration in this study was demonstrated to be neuroprotective (135).

In AIE the seizures are likely driven by the combination of ongoing neuroinflammation as well as alterations in neuronal excitability set points due to antibody effects on receptors. For example, the ability of the NMDAR antibody to generate seizures in animal models is controversial. In a study by Wright et al. purified NMDAR antibodies from patients injected into the brains of mice are able to lower seizure threshold, but spontaneous seizures are not seen on continuous EEG recordings (136). Conversely a more recent study by Taraschenko et al. demonstrated the generation of spontaneous non-convulsive seizures on continuous EEG monitoring in mice injected with rabbit Anti-NMDAR IgG or patient CSF compared with a control group (137). Interestingly in this second study the mice injected with patient CSF had 4–5 fold more seizures than the group injected with rabbit Anti-NMDAR IgG (137). It is plausible that the addition of pro-inflammatory compounds present in the CSF, such as IL-1β, in the setting of lower excitability thresholds, could drive epileptogenesis in AIE.

Microglial activation and proliferation may also contribute to long-term cognitive changes seen in patients with AIE. In NMDAR antibody associated AIE, >75% of patients are reported to have cognitive impairment of some degree as part of their illness, while 76% have cognitive impairment persisting beyond the acute illness (138). While this is largely thought to be mediated by antibodies targeting important neuronal receptors, it is unclear why these deficits should persist beyond the acute illness. While the cellular mechanisms for ongoing cognitive dysfunction have not been examined in AIE, there is similarity with another antibody-associated condition which can affect the CNS and cause cognitive dysfunction, Systemic Lupus Erythematous (SLE). In SLE patients can develop antibodies to the NMDAR GluN2A and GluN2B subunits (139). These patients manifest deficits in executive function, processing speed and memory even after the antibodies have been cleared from the CNS. This is postulated to occur through ongoing structural and functional changes mediated by microglia (139), which appear to occur in an antibody independent manner.

Interestingly ongoing cognitive dysfunction in AIE can have structural correlates in neuroimaging. In LGI-1 antibody associated AIE, cognitive dysfunction correlated with putamenal atrophy as well as changes on diffusion tensor imaging in the white matter tracts of the anterior corona radiate, anterior internal capsule and anterior third of the corpus callosum (140). It remains unclear whether these structural and functional changes are driven by the auto-antibodies or by microglial and monocyte driven neuroinflammation as suggested in SLE.

## Future Directions

While there is certainly some evidence to suggest an important role for the innate immune system in AIE, this area has generally been overlooked in favor of the adaptive immune system resulting in a paucity of research in this area. Immunophenotyping studies focusing on innate components, more detailed cytokine and cell transcriptome analyses, and further epidemiological studies examining associations with other pro-inflammatory states/first-hit events will contribute to building knowledge in this area. Given the potential for the innate response to be a conserved pathway across the subtypes of AIE, an understanding of the role it plays may lead to the detection and use of common biomarkers across different subtypes of AIE. This would be particularly helpful in seronegative AIE.

Furthermore, given the high prevalence of seizures in AIE and the likelihood that this relates to the CNS pro-inflammatory state, further investigation into these components may also provide added understanding of a potential pathway of epileptogenesis and the repurposing of targeted immunotherapy such as IL-6 blockade in certain types of epilepsy.

Finally a greater understanding of the role for innate immune pathways in AIE may provide additional treatment options. This could include targeting important molecules involved in innate cell recruitment and activation such as IL-1β, TLR4, and CCL2. Anakinra is an existing IL-1R blocking monoclonal antibody which has been used previously in a microglia predominant neuroinflammatory disorder (141). CCL2 blockade targeting myeloid cell infiltration has been successful in animal models of human cancers (142). There are also a number of promising TLR4 antagonists that have been successful in treating inflammatory disease in pre-clinical trials, although none have been successful in clinical trials as yet (143, 144). Recent advances in treatment in other CNS autoimmune disorders may also be re-purposed for AIE. These include Eculizumab, a monoclonal antibody targeting the complement cascade, and Inebilizumab, a monoclonal antibody targeting CD19 expressing cells. Another therapy Satralizumab, an antibody targeting the IL-6R, has the most potential to be converted into therapy for AIE, given the potential role of IL-6 in the pro-inflammatory cascade and the success with Tocilizumab.

## Conclusion

While a number of the important interactions between the innate, adaptive and neural components in CNS autoimmunity and neuroinflammation have been well-studied, there remains significant gaps in our knowledge. AIE provides a unique disorder which can assist us in understanding the mechanisms of CNS autoimmunity and its genesis. In particular the role of dysregulated innate cell activity in driving autoreactive lymphocyte proliferation and maturation to immunoreactive lymphocytes. This will also provide us with potential improvements in diagnosis and treatment of AIE, as well as other CNS autoimmune diseases.

## Author Contributions

RW performed the literature search and wrote the manuscript. MM, TO'B, HB, DT, and KB oversaw preparation of the manuscript, and contributed to writing and editing of the manuscript.

### Conflict of Interest Statement

The authors declare that the research was conducted in the absence of any commercial or financial relationships that could be construed as a potential conflict of interest.
